# Clinical, Psychological, and Social Determinants of Brace Compliance in Adolescent Idiopathic Scoliosis: A Systematic Review and Meta-Analysis

**DOI:** 10.3390/jfmk11010068

**Published:** 2026-02-08

**Authors:** Marco Sapienza, Marco Simone Vaccalluzzo, Emanuele Perricone, Carmelo Giannone, Alessia Caldaci, Giuseppe Musumeci, Andrea Vescio, Gianluca Testa, Vito Pavone

**Affiliations:** 1 Department of General Surgery and Medical Surgical Specialties, Section of Orthopaedics, A.O.U. Policlinico Rodolico-San Marco, University of Catania, 95123 Catania, Italy; sapienza.marco@studium.unict.it (M.S.); marcovaccalluzzo@hotmail.it (M.S.V.); emanuele.perricone14@gmail.com (E.P.); carmelo.giannone32@gmail.com (C.G.); alessia.c.92@hotmail.it (A.C.); 2 Department of Biomedical and Biotechnological Sciences, Section of Anatomy, Histology and Movement Science, School of Medicine, University of Catania, Via S. Sofia No.97, 95123 Catania, Italy; g.musumeci@unict.it; 3 Research Center on Motor Activities (CRAM), University of Catania, Via S. Sofia No.97, 95123 Catania, Italy; 4 Department of Life Science, Health, and Health Professions, Link Campus University, 00165 Rome, Italy; andreavescio88@gmail.com; 5 Department of Orthopaedic and Trauma Surgery, “Mater Domini” University Hospital, “Magna Græcia” University, 88100 Catanzaro, Italy

**Keywords:** scoliosis, adolescent, orthotic devices, patient compliance, monitoring, physiologic, quality of life

## Abstract

**Background:** Brace adherence is a key determinant of treatment success in adolescents with idiopathic scoliosis. However, adherence is influenced by multiple clinical, psychological, and social factors, and reported wear times vary widely across studies. This systematic review and meta-analysis aimed to identify determinants of brace adherence and assess their quantitative impact on real wear. **Methods:** A comprehensive search was conducted in PubMed/MEDLINE, Scopus, Web of Science, Embase, and Google Scholar from database inception to November 2025. A total of 1040 records were identified, 620 were screened, and 45 full-text articles were assessed for eligibility. In total, 17 studies met the inclusion criteria and were included in the qualitative synthesis, and 10 provided extractable quantitative data and were included in the meta-analysis. A random-effects model was used to calculate pooled mean differences for identified determinants, including sex, age, early adherence, and sensor-based monitoring. **Results:** In total, 17 studies involving 1716 adolescents were included, and 10 provided extractable quantitative data for meta-analysis. Objective sensor-based monitoring was consistently associated with higher adherence, with a pooled mean difference of 25.6 percent compared with non-sensor methods. Early adherence significantly predicted long-term compliance, with a mean difference of 9.6 percent. Younger adolescents demonstrated greater adherence than older patients, with a mean difference of 19.1 percent, while sex differences favored females but did not reach statistical significance. Psychosocial determinants such as body image perception, stress, family dynamics, and religious environment played an important role in modulating adherence. Higher body mass index (BMI) and reduced quality of life were associated with poorer compliance. Overall, studies evaluating positive determinants reported a pooled mean adherence of 89.6 percent compared with 67.7 percent in studies characterized by negative determinants. **Conclusions:** Brace adherence is determined by a combination of clinical and psychosocial factors. Sensor-based monitoring, strong early adherence, and supportive environments consistently enhance compliance, whereas stress, poor body image, and higher BMI hinder wear. Targeted interventions, early counseling, and standardized adherence metrics are needed to improve outcomes in brace-treated scoliosis.

## 1. Introduction

Adolescent idiopathic scoliosis (AIS) is the most common spinal deformity in otherwise healthy adolescents, with a prevalence between 0.5% and 5% and notable geographic and sex-related variability [1,2]. Curve progression during growth can lead to cosmetic deformity, pain, and long-term functional impairment, making early identification and management essential [3]. Bracing is the cornerstone of conservative treatment for moderate AIS in skeletally immature patients. Its effectiveness in preventing curve progression is closely linked to adherence, and higher daily brace-wearing times are associated with improved outcomes [4,5]. Conventional self-reported adherence measures tend to overestimate true wear time, whereas objective monitoring through temperature- or pressure-based sensors has shown significantly lower actual compliance [6,7,8]. Recent studies have also shown that objective monitoring may itself enhance adherence through increased patient awareness and accountability [9,10].

A growing body of evidence indicates that brace compliance is influenced by clinical, psychological, and social variables. High stress levels, negative body image, and reduced quality of life are associated with lower adherence rates [11,12], whereas structured counseling, family support, and favorable psychosocial environments appear to promote better compliance [13,14]. Demographic factors may also play a role: several studies report higher adherence among younger adolescents and female patients [15,16,17].

Despite the increasing number of studies on determinants of brace adherence, the evidence remains heterogeneous due to differences in study design, compliance definitions, and measurement methodologies. To date, no comprehensive synthesis has quantitatively compared the relative impact of positive and negative influencing factors. The aim of this systematic review and meta-analysis was to identify and quantify the clinical, psychological, and social determinants of brace compliance in the context of AIS to provide clinicians with evidence-based insights to optimize conservative management strategies.

## 2. Materials and Methods

### 2.1. Study Design

This study was conducted to evaluate the clinical, psychological, and social determinants of brace adherence among adolescents with idiopathic scoliosis. The study adhered to the PRISMA 2020 guidelines and was registered on the International Prospective Register of Systematic Reviews (PROSPERO) (registration number: CRD420251252699). Detailed information regarding the PRISMA checklist is available in the Supplementary Materials.

### 2.2. Search Strategy

A comprehensive literature search was performed on PubMed/MEDLINE, Scopus, Web of Science Core Collection, and Embase. To ensure completeness, the first 300 entries returned by Google Scholar were also screened for additional relevant studies. Searches were conducted from database inception to November 2025. The search strategy combined controlled vocabulary (MeSH terms in PubMed and EMTREE in Embase) with free-text keywords related to adolescent idiopathic scoliosis, brace treatment, adherence, compliance, wear time, and monitoring systems. A representative PubMed search string included the terms “adolescent idiopathic scoliosis”, “brace” or “orthosis”, and “adherence” or “brace wear” or “monitoring”. Search strategies were adapted for each database to account for differences in syntax and indexing. Reference lists of all included studies and relevant reviews were screened to identify additional publications. No restrictions regarding language, publication date, or geographic setting were applied.

### 2.3. Eligibility Criteria and Outcomes of Interest

Studies were included if they evaluated adolescents with idiopathic scoliosis treated with a rigid thoracolumbosacral orthosis or an equivalent brace and if they reported quantitative measures of adherence, such as daily hours of wear, percentage of prescribed hours, or sensor-derived measurements. Eligible study designs included prospective and retrospective observational studies, interventional studies, and cohort analyses published in English as full-text articles. Studies were excluded if they focused on congenital, neuromuscular, or syndromic scoliosis; if they lacked extractable adherence data; or if they were qualitative-only reports, conference abstracts, editorials, or expert commentaries.

The primary outcome of this systematic review was brace compliance, which was defined as the proportion of prescribed brace-wear hours effectively completed by adolescents undergoing orthotic treatment for idiopathic scoliosis. Whenever available, adherence was recorded as the mean percentage of wear time or as the absolute number of hours worn per day. The secondary outcomes were related to the variation in compliance in clinical and psychosocial subgroups. In particular, the review examined differences associated with the method used to measure adherence (objective sensor-based monitoring versus self-reported wear), the level of adherence achieved during the early phase of treatment, patient age, and sex. Additional determinants were also evaluated when reported, including psychological factors such as stress, body image perception, quality of life, and family dynamics, as well as clinical factors such as body mass index (BMI) and brace characteristics. For each study, mean values, standard deviations, and effect estimates were extracted to allow for qualitative comparison and quantitative synthesis when appropriate.

### 2.4. Study Selection

All retrieved records were imported into reference management software for the removal of duplicates. Two independent reviewers screened titles and abstracts to identify potentially relevant articles. The full texts were obtained when eligibility could not be determined from the abstract alone. Disagreements were resolved through discussion until consensus was reached.

**Figure 1:**
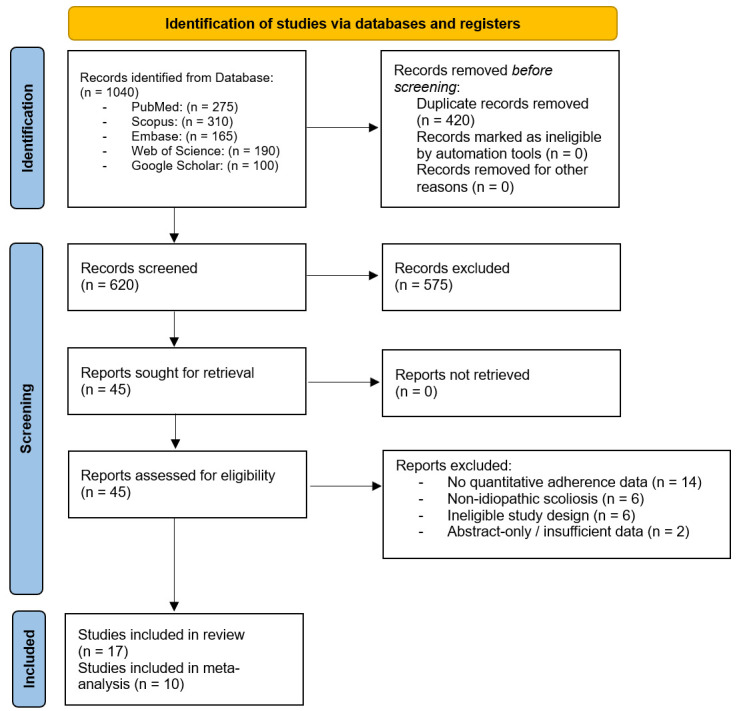
PRISMA 2020 flow diagram summarizing the study selection process, including database identification, removal of duplicate records, title and abstract screening, full-text eligibility assessment, and final inclusion of 17 studies in the qualitative synthesis and 10 studies in the meta-analysis.

Studies meeting all inclusion criteria were included in the qualitative synthesis. Only studies providing extractable numerical adherence data (means, standard deviations, and sample sizes) were considered eligible for quantitative synthesis and were therefore incorporated into the meta-analysis. The selection process is illustrated in the PRISMA flow diagram in Figure 1.

### 2.5. Data Extraction

Data extraction was performed using a predefined form to ensure consistency. The extracted variables included study characteristics (year, country, design, and sample size), patient demographics (age and sex distribution), baseline clinical data (Cobb angle and skeletal maturity), and details about the brace prescription, such as the type of orthosis, recommended daily wear time, and duration of follow-up. Adherence metrics were collected in the form in which they were reported, with preference given to objective sensor-based measurements when both objective and subjective data were available. Information was documented in regard to the determinants of adherence investigated in each study, as well as any correlation coefficients, between-group differences, or associated outcomes. When necessary, adherence data were converted or standardized according to accepted meta-analytic procedures.

### 2.6. Quality Assessment

The methodological quality of the included studies was assessed using the Newcastle–Ottawa Scale (NOS). The NOS is a validated tool for evaluating observational research in three domains: selection (0–4 points), comparability (0–2 points), and outcome assessment (0–3 points), with a maximum score of 9. Qualitative methodological aspects relevant to adherence research were also considered, including the reliability of adherence measurement, control of potential confounders, outcome reporting, and the adequacy of follow-up.

### 2.7. Definition and Standardization of Adherence

Adherence definitions varied considerably between studies, so the outcomes were standardized to daily hours of brace wear and the percentage of prescribed wear. Sensor-based adherence obtained through temperature or pressure sensors embedded in the brace was considered the most accurate measure. When only subjective reports were available, the patients’ or parents’ diaries and interviews were used. When required, statistical transformations were used to obtain the mean and standard deviation values from the medians, ranges, or interquartile ranges.

### 2.8. Statistical Analysis

Quantitative synthesis was performed using a random-effects model (DerSimonian–Laird) to account for expected clinical and methodological heterogeneity between studies. Effect sizes were expressed as mean differences for continuous outcomes and as odds ratios for categorical variables. Statistical heterogeneity was quantified using the *I*^2^ statistic. Prespecified subgroup analyses were performed to explore differences based on age, sex, early versus late adherence, objective versus subjective adherence measurement, and the broader categorization of positive versus negative determinants. For the age subgroup analysis, ‘younger’ adolescents were defined as patients aged ≤ 13 years, while ‘older’ adolescents were defined as those aged ≥ 14 years, based on the most commonly reported cut-offs across the included studies [6,15,16,17]. Publication bias was assessed qualitatively due to the limited number of studies per subgroup. All analyses were conducted in accordance with the PRISMA recommendations and standard meta-analysis procedures. All pooled estimates are reported as mean differences (MD), not standardized mean differences (SMD), because adherence outcomes were measured on comparable continuous scales across studies (percentage of prescribed wear or hours/day).

## 3. Results

### 3.1. Study Selection

The database search identified 1040 records (275 from PubMed, 310 from Scopus, 190 from Web of Science, 165 from Embase, and 100 from Google Scholar). After removal of 420 duplicates, 620 records remained for title and abstract screening. Of these, 575 were excluded because they did not meet the inclusion criteria.

In total, 45 full-text articles were assessed for eligibility. In total, 28 studies were excluded for the following reasons: absence of extractable quantitative adherence data (*n* = 14), non-idiopathic scoliosis populations (*n* = 6), ineligible study design such as qualitative-only reports or narrative reviews (*n* = 6), and conference abstracts without sufficient data (*n* = 2). In total, 17 studies fulfilled all inclusion criteria and were included in the qualitative synthesis. In total, 10 of these studies provided suitable numerical adherence data for pooling and were incorporated into the meta-analysis.

### 3.2. Study Characteristics

The 17 included studies evaluated 1716 adolescents with idiopathic scoliosis. As shown in Table 1, the mean age ranged from 8 to 16 years, and there was a clear predominance of female patients. The baseline Cobb angles varied between 15° and 53°, and most patients were skeletally immature (Risser 0–2). The brace prescriptions were relatively homogeneous, and the average recommended wear time was approximately 20 h per day, although the definition and measurement of actual adherence differed substantially between studies. Six studies used objective electronic sensors (temperature or force) to record brace use, whereas the remaining 11 relied on self-reported methods such as diaries, interviews, or questionnaires. The mean follow-up duration was about 9 months, although some prospective cohorts extended beyond one year.

### 3.3. Determinants of Brace Compliance

#### 3.3.1. Positive Factors

- Use of sensors

**Figure 2:**
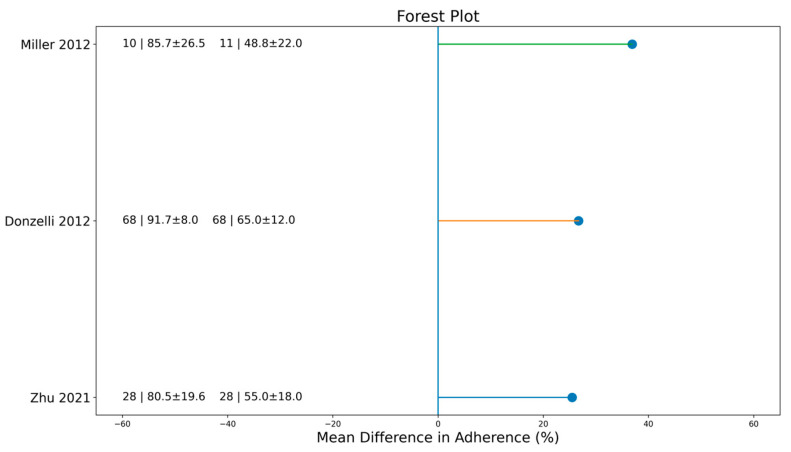
Forest plot comparing brace adherence between patients monitored with sensors and those assessed with standard methods. Values represent mean differences (hours of brace wear per day) with 95% confidence intervals. Random-effects model [7,8,10].

Objective monitoring emerged as one of the strongest positive determinants of brace adherence. In a randomized study by Miller, patients who were informed about the presence of a temperature-based sensor wore their brace 5.24 h per day more than uninformed controls, and compliance increased from 48.8% to 84.2% during the first treatment week (*p* = 0.022). Zhu reported a mean wear time of 14.1 ± 2.9 h/day and a quality compliance of 64.8 ± 22.2%, while Donzelli observed a median wear time of 21 h/day, which corresponded to 91.7% adherence for a 23 h prescription. In all sensor studies, objective measurements systematically revealed higher actual adherence and reduced overestimation compared with self-report, as shown in Table 1 and Figure 2.

- Early adherence

**Figure 3:**
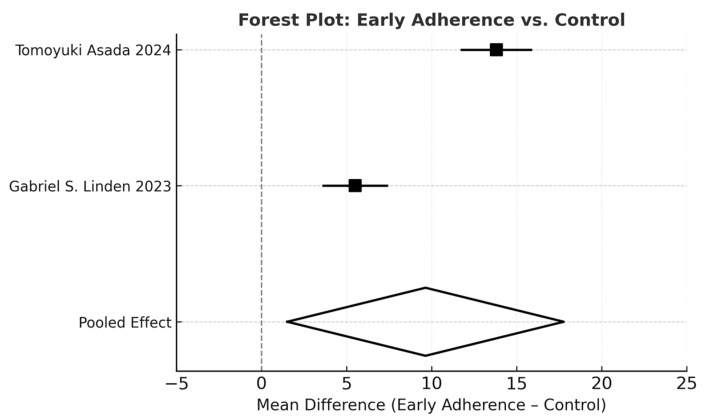
Forest plot evaluating the effect of early adherence on brace wear. Patients who established adherence early in treatment demonstrated significantly higher compliance than controls. Mean differences and 95% confidence intervals were estimated using a random-effects model [9,18].

Two prospective cohorts showed that adherence during the first month of treatment is highly predictive of subsequent compliance. Asada demonstrated that early adherence independently predicted optimal brace wear at 6 months (odds ratio (OR) 1.52; 95% confidence interval (CI) 1.30–1.79; *p* < 0.001). Linden reported that initially adherent patients maintained approximately 5 additional hours of brace wear per day at 6, 12, and 24 months. The meta-analysis confirmed this effect, with a pooled difference of +9.64% in favor of early-adherent patients (Figure 3).

- Sex

Several studies reported higher adherence among female adolescents. Fregna found a compliance of 91% among females compared with 84% among males, and Takemitsu observed a similar trend (77.2% vs. 54.7%). Linden showed that female patients had between 8.5- and 16.6-fold higher odds of maintaining adherence at different time points during follow-up.

- Age

Younger age was consistently associated with better compliance. In Fregna’s cohort, adolescents aged 10–13 years achieved a mean adherence of 92% compared with 88% for those aged 14–16 years. DiRaimondo reported 87% adherence among grade-school children and 63% among high-school students, whereas Takemitsu reported a decline from 84% at the age of 10 years to 60% at the age of 14 years. Hasler similarly found higher adherence among younger patients (72% vs. 47%).

- Counseling

Structured counseling interventions led to significant improvements in adherence. In a randomized study by Karol, patients receiving counseling based on objective feedback wore their brace 13.8 h/day, while 10.8 h/day was observed in the control group (*p* = 0.002). Over the first 180 days, counseled patients maintained a higher average wear (15.0 vs. 12.5 h/day) and had a lower rate of curve progression ≥ 6° (46% vs. 59%).

- Mindfulness-based intervention (MBrace)

Li evaluated a mindfulness-based program (MBrace) and found a short-term benefit in brace wear. At 2 months, adolescents in the MBrace group wore the brace 1.89 h/day more than controls (*p* = 0.02). At 8 months, a difference remained (1.64 h/day; *p* = 0.09) and became statistically significant after excluding outliers (2.27 h/day; *p* = 0.01), suggesting a persistent positive effect on adherence.

- BMI and psychosocial support

BMI showed a graded impact on adherence. In Karol’s series, underweight patients wore the brace for a mean of 15.7 h/day, normal-weight patients wore the brace for 12.5 h/day, overweight patients wore it for 11.7 h/day, and obese patients wore it for 9.0 h/day. These results correspond to the estimated compliance ranging from approximately 87% to 50%. Psychosocial factors also contributed: in Gornitzky’s study, higher Brace-Favorable Factor Scores were associated with adherence above 75%, and adolescents from highly religious families achieved a median compliance of 98% compared with 76% in less religious families (*p* = 0.008).

#### 3.3.2. Negative Factors

- Trunk appearance, body image, and older age

**Figure 4:**
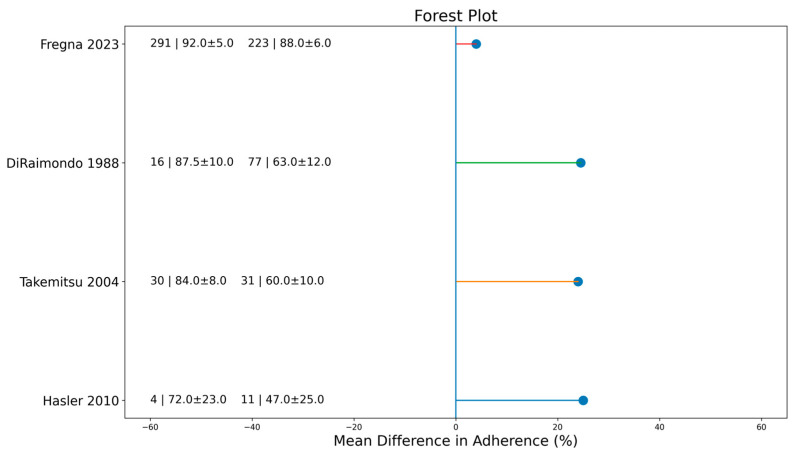
Forest plot evaluating the effect of patient age on brace compliance. Mean differences (younger vs. older adolescents) are shown with 95% confidence intervals using a random-effects model. Younger adolescents (≤13 years) versus older adolescents (≥14 years) [6,15,16,17].

Chan reported that poor perception of trunk appearance negatively affected adherence. The least compliant group showed smaller improvements in the Trunk Appearance Perception Scale (TAPS) (−0.70) and a marked reduction in Brace Questionnaire (BrQ) scores (−16.28). Many patients limited brace wear to nighttime in order to reduce social exposure. Fregna, DiRaimondo, Takemitsu, and Hasler observed an age-related decline in adherence. They indicated that older adolescents were less likely to maintain prescribed brace wear, which reinforces age as a negative determinant of compliance (Figure 4).

- Stress and quality of life

Moderate stress levels were associated with reduced adherence. In Asada’s cohort, the moderate-stress group’s mean compliance was 65.2% and was particularly lower during the daytime and weekends. Rivett similarly found that non-compliant patients wore their brace for 14.4 h/day compared with 21.4 h/day among compliant patients. Non-compliant patients exhibited significantly lower BrQ scores, indicating that poorer quality of life is closely linked to decreased adherence.

- Overweight and obesity

Overweight and obese adolescents showed the lowest adherence. In Karol’s study, compliance fell to around 65% in overweight patients and 50% in obese patients. Discomfort, heat intolerance, and mechanical difficulties while wearing the brace likely contributed to this reduction.

#### 3.3.3. Neutral Factors

Some variables showed no consistent association with brace adherence and were considered neutral determinants. These included the number of prescribed hours per day, general family functioning, parenting style, and the presence of routine family conflicts. In some cohorts [18,19,21], stress and quality-of-life measures did not correlate significantly with actual wear, suggesting that their impact may be context- or population-dependent.

### 3.4. Meta-Analysis

**Figure 5:**
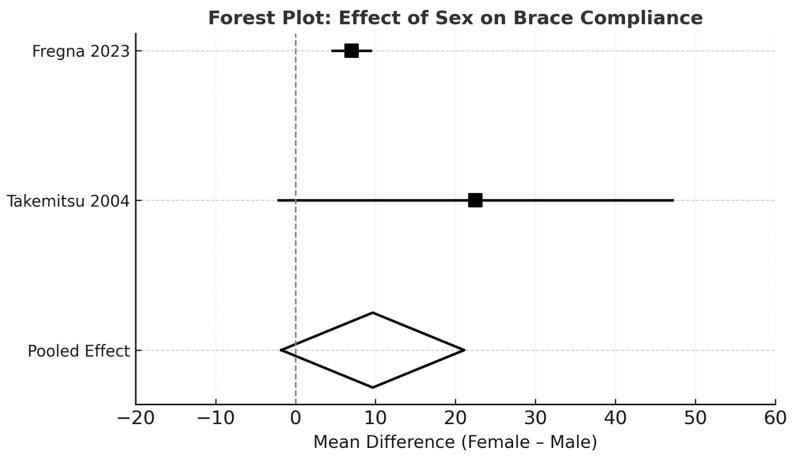
Forest plot comparing brace compliance between female and male patients. Mean differences and 95% confidence intervals were calculated using a random-effects model [6,17].

The random-effects meta-analysis confirmed the role of several determinants. Younger adolescents showed significantly higher adherence than older ones, with a pooled mean difference of 19.14% (95% CI 3.68–34.59; *p* = 0.02; Figure 4). Early adherence was also associated with improved compliance, with a pooled difference of +9.64% (95% CI 1.51–17.77; *p* = 0.02; Figure 3). Although most individual studies reported higher adherence among female patients, the pooled effect for sex did not reach statistical significance (*p* = 0.10; *I*^2^ = 33%; Figure 5). In contrast, sensor use produced the largest and most consistent effect, with a pooled difference of 25.56% in favor of sensor-monitored patients (95% CI 12.03–39.09; *p* = 0.0002; *I*^2^ = 19%; Figure 2).

When studies were grouped according to the direction of the investigated determinant, the pooled mean adherence of 89.6% among those evaluating positive factors (such as younger age, early adherence, female sex, sensor monitoring, favorable BMI, counseling, and supportive family environment). However, the pooled mean adherence was 67.7% for studies focusing on negative factors (older age, moderate stress, low quality of life, and overweight/obesity). The difference between these groups was highly significant (*p* < 0.0001), which underscores the substantial impact of modifiable clinical, psychological, and social determinants on brace-wearing behavior. A detailed summary of positive and negative determinants of brace adherence, including mean compliance values where available, is reported in Table 2.

## 4. Discussion

This systematic review and meta-analysis demonstrates that brace adherence in AIS results from the interplay of clinical, technological, psychological, and social determinants. Our findings reinforce the multifactorial perspective proposed in contemporary AIS management frameworks and in the Society on Scoliosis Orthopaedic and Rehabilitation Treatment 2016 guidelines, which highlight that bracing effectiveness depends not only on biomechanical correction, but also on behavioral, emotional, and environmental influences [22]. The strong impact of early adherence, objective monitoring systems, psychological distress, and family support aligns with literature indicating that brace treatment is as much a behavioral intervention as a biomechanical one [4,23].

### 4.1. Objective Monitoring Systems

Several included studies confirmed the limitations of self-reported brace wear, which consistently overestimates true adherence. Sensor-based monitoring (temperature, pressure, or accelerometry) provides more accurate measurements and, importantly, may improve adherence through mechanisms of accountability and behavioral reinforcement [7,8]. Similar improvements with digital monitoring have been reported for other pediatric chronic conditions, in which real-time feedback increases treatment compliance [24,25,26]. Recent innovations, including smartphone-linked apps, cloud dashboards, and smart braces, suggest that adherence may soon become a continuously monitored parameter. These systems are consistent with emerging digital-health strategies that enable real-time communication, personalized alerts, and adaptive clinical decision-making. Such tools may ultimately integrate brace treatment into a broader digital therapeutic ecosystem.

### 4.2. Early Adherence and the Initial Adaptation Phase

Our analysis underscores that adherence during the first month of treatment is strongly predictive of long-term compliance. This phenomenon aligns with behavioral models such as the Health Belief Model and Self-Determination Theory, which indicate that early perceptions of benefit, comfort, and feasibility shape subsequent engagement [27,28]. The Scoliosis Research Society recommendations and other bracing protocols emphasize the importance of early education, rapid brace optimization, and close follow-up during the initial adaptation period [29,30]. Therefore, early adherence represents both a prognostic marker and a therapeutic window during which targeted interventions may significantly alter long-term adherence trajectories.

### 4.3. Age and Sex Differences

Several studies demonstrated higher brace adherence among younger adolescents than older ones, likely due to closer parental supervision, greater treatment acceptance, and fewer social constraints. Fregna et al. [17], DiRaimondo and Green [15], Takemitsu et al. [6], and Hasler et al. [16] all reported age-related declines in adherence. Sex-related differences were less consistent: while multiple studies observed higher adherence in female adolescents, the pooled effect did not reach statistical significance. This variability may reflect population-specific psychosocial factors. Reichel and Schanz [31] noted that girls often demonstrate heightened attention to body image, whereas boys may encounter social or athletic barriers that reduce daytime brace use. Overall, although trends exist, the heterogeneity between cohorts suggests a need for additional standardized comparative studies.

### 4.4. Psychological Determinants: Body Image, Stress, and Quality of Life

Psychological well-being plays a central role in adherence. Poor body image, stress, and reduced quality of life were associated with lower adherence in several studies. Chan et al. [12] reported that negative trunk appearance perception markedly reduced daily brace wear, while Rivett et al. [11] demonstrated that non-compliant patients had significantly lower BrQ scores. These findings echo broader psychosocial literature indicating that adolescents with visible medical devices frequently experience embarrassment, teasing, avoidance behaviors, and social withdrawal, which are all factors that reduce daytime brace use. Cognitive-behavioral approaches, as shown by Negrini et al. [32], can improve brace acceptance and mitigate emotional resistance, which supports the integration of psychological screening and targeted counseling into bracing programs.

### 4.5. Social and Family Environment

Family dynamics represent one of the strongest determinants of treatment adherence. Gornitzky et al. [14] showed that structured family routines, parental monitoring, and emotional support significantly increase adherence. Evidence from pediatric chronic disease management further reinforces the impact of parental involvement, shared responsibility, and consistent communication on treatment consistency [33]. MacLean et al. [34] demonstrated that families who receive structured coping strategies show better adaptation and brace acceptance. Consequently, the SOSORT guidelines emphasize that parents should be considered active therapeutic partners rather than passive observers [22]. Therefore, ensuring systematic family engagement may be essential to improving real-world adherence outcomes.

### 4.6. Brace Design, Fit, Comfort, and BMI

Comfort and brace ergonomics substantially influence adherence. Overweight and obese adolescents often experience poorer fit, increased heat accumulation, and higher friction at the brace–skin interface, which contribute to reduced wear time [12]. Karol et al. [13] reported markedly lower adherence among overweight and obese patients.

From a biomechanical standpoint, pressure distribution, padding configuration, and heat dissipation have been shown to affect brace tolerance [35]. Zhu et al. [10] demonstrated that temperature build-up correlates with discomfort and wear variability. Emerging 3D-printed brace technologies may improve comfort through individualized pressure optimization and improved ventilation. Preliminary data from Li et al. [19] suggest that there are potential benefits, although further research is needed to evaluate the effects on long-term adherence.

### 4.7. Alignment with International Clinical Guidelines

The findings of this review align closely with the SOSORT 2016 guidelines, which emphasize individualized patient education, early adaptation strategies, psychosocial assessment, and the use of objective monitoring to optimize bracing outcomes [22]. Furthermore, the SRS BrAIST trial demonstrated a clear dose–response relationship between hours of brace wear and treatment success, which reinforces that adherence is the primary modifiable factor determining curve progression [4]. Together, these frameworks support a patient-centered approach that addresses not only mechanical correction but also behavioral adherence dynamics.

### 4.8. Digital Tools, Artificial Intelligence, and Future Technologies

Digital technologies are reshaping the monitoring and management of adherence. Sensor-based systems are the most validated tools for capturing real-time brace wear, and awareness of being monitored may enhance adherence [7,36]. Early feasibility studies of smartphone-linked monitoring platforms indicate good acceptability among families and potential improvements in adherence patterns [10]. Machine-learning approaches have recently been explored to predict non-adherence by analyzing temporal wear patterns and are opening up the possibility of anticipatory interventions. As telemedicine and remote monitoring platforms expand, digitally integrated bracing protocols may become standard practice in AIS management [37].

### 4.9. Clinical Implications

The evidence synthesized in this review highlights that optimizing brace adherence requires a comprehensive multimodal strategy that combines technological, psychological, and family-centered interventions. Adopting such an approach may meaningfully increase real-world adherence, improve treatment effectiveness, and reduce progression requiring surgery. Clinically, this includes:

- early optimization of brace fit and comfort;
- structured early adaptation protocols;
- routine psychological assessment and counseling;
- active parental participation;
- integration of objective monitoring systems.

### 4.10. Strengths and Limitations

This review adhered to the PRISMA standards and integrated both qualitative and quantitative evidence. Its strengths include the use of predefined extraction criteria, the incorporation of objective adherence data, and comprehensive evaluation of psychosocial determinants. Its limitations include heterogeneity in adherence definitions, reliance on observational designs, variable psychological assessment tools, and inconsistent reporting of brace design parameters. Cultural and environmental differences between countries may also contribute to heterogeneity, but were not consistently documented in different studies. Despite these limitations, the reproducibility of key findings supports the robustness of the identified determinants, particularly in regard to early adherence, family involvement, and objective monitoring.

## 5. Conclusions

This systematic review and meta-analysis have shown that brace adherence in adolescents with idiopathic scoliosis is strongly influenced by multiple clinical and psychosocial factors. Objective monitoring systems consistently yielded higher and more accurate adherence than self-reported methods, which supports their integration into routine care. Early adherence proved to be a decisive predictor of long-term wear, while younger age and supportive family environments were associated with better compliance. Conversely, negative body image, stress, reduced quality of life, and higher BMI were linked to poorer adherence. Heterogeneity among studies limits the strength of some comparisons, but the overall consistency of findings highlights the need for early counseling, psychosocial support, and tailored interventions for at-risk patients. Future studies should adopt standardized adherence metrics and evaluate targeted strategies aimed at improving brace wear and treatment outcomes.

## Figures and Tables

**Table 1:** Characteristics of the included studies.

Author (Year)	Total Patients	Mean Age	Age Range	Cobb Angle Range	Risser Stage	Male	Female	Follow-Up (Months)	Brace Prescription (h/day)
Asada et al. [18]	122	12.6	10–15	20–40	–	8	114	6	20
Zhu et al. [10]	28	12.4	10–15	25–40	0–2	5	21	6	23
Chan et al. [12]	42	12.6	11–15	25–40	0–2	0	42	–	–
DiRaimondo et al. [15]	76	13.3	8–15	25–40	0–2	0	–	–	23
Linden et al. [9]	60	12.5	10–14.8	–	–	10	50	–	18
Karol et al. [13]	171	12.3	10–16	25–40	0–2	6	165	–	–
Li et al. [19]	83	13.5	10–15	15–30	–	21	62	8	18
Miller et al. [7]	21	12.4	8–15	20–50	0–2	5	16	5	18
Asada et al. [20]	41	12.4	10–15	25–45	0–4	3	38	22	20
Donzelli et al. [8]	68	14.5	12–16	35–53	–	15	53	5	23
Schwieger et al. [21]	167	11.7	–	20–40	–	0	167	–	18
Fregna et al. [17]	514	13.8	12–15	25–45	0–4	90	424	–	22
Karol et al. [13]	175	12.3	10–14	25–45	0–2	16	159	–	19.5
Rivett et al. [11]	31	14.5	13–16	25–40	–	0	31	–	23
Hasler et al. [16]	15	13.1	11–15	20–43	0–3	0	15	–	23
Takemitsu et al. [6]	61	12.0	6–16	20–45	0–2	7	54	17	23
Gornitzky et al. [14]	41	12.5	11–14	26–36	–	4	37	–	18
Total	1716	12.8	8–16	15–53	0–4	190	1448	9	20.0

Values refer to baseline characteristics reported by each study. “–” indicates that the variable was not reported in the original article. Age, age range, Cobb angle range, Risser stage, follow-up duration, and brace prescription hours are presented exactly as provided by the authors. Total sex distribution (Male, Female) reflects the number of patients included in each study. The “Total” row summarizes cumulative values across all included studies.

**Table 2:** Summary of positive and negative determinants of brace adherence. Overview of all clinical, psychological, and social determinants associated with brace adherence reported across the included studies. Mean compliance values, standard deviations, and sample sizes are shown when available. Positive determinants refer to variables associated with increased adherence, whereas negative determinants indicate reduced adherence.

Author (Year)	Determinant Type	Factor	Mean Compliance (%)	SD	Sample Size
Linden (2023) [9]	Positive	Female sex	–	–	–
Li (2023) [19]	Positive	MBrace system	–	–	–
Gornitzky (2023) [14]	Positive	Religious family	98	–	6
Linden (2023) [9]	Positive	Early adherence	93.9	9.7	32
Asada (2024) [20]	Positive	Early adherence	92.2	1.5	72
Fregna (2023) [17]	Positive	Early age	92	5	291
Donzelli (2012) [8]	Positive	Sensors	91.7	–	68
Fregna (2023) [17]	Positive	Female sex	91	15.5	424
DiRaimondo (1988) [15]	Positive	Early age	87.5	10	16
Miller (2012) [7]	Positive	Sensors	85.7	26.5	10
Takemitsu (2004) [6]	Positive	Early age	84	8	–
Karol (2016) [13]	Positive	Low BMI	80	5	21
Zhu (2021) [10]	Positive	Sensors	80.5	19.6	28
Takemitsu (2004) [6]	Positive	Female sex	77.2	–	54
Karol (2016) [13]	Positive	Counseling	76.67	8.9	93
Hasler (2010) [16]	Positive	Early age	72	23	4
Chan (2014) [12]	Negative	TAPS (poor appearance perception)	–	–	–
Fregna (2023) [17]	Negative	Older age (>14 years)	88	–	223
Asada (2023) [20]	Negative	Moderate stress	65.2	7.1	14
DiRaimondo (1988) [15]	Negative	Older age	63	–	77
Rivett (2009) [11]	Negative	Low quality of life	62	7.6	11
Karol (2019) [13]	Negative	Overweight/obesity	60.5	5.8	30
**Pooled Mean–Positive Factors**			89.6	–	–
**Pooled Mean–Negative Factors**			67.7	–	–

Mean compliance, standard deviation, and sample size are reported when available. “–” indicates that the original study did not provide the corresponding numerical value. Positive determinants refer to factors associated with higher adherence, while negative determinants reflect factors associated with reduced adherence. Pooled means for positive and negative determinants were calculated from studies with extractable quantitative data.

## Data Availability

All data extracted and analyzed in this review are derived from previously published studies. Additional information is available from the corresponding authors upon reasonable request.

## Supplementary Materials

The following supporting information can be downloaded at: https://www.mdpi.com/article/10.3390/jfmk11010068/s1. Ref. [38] is cited in Supplementary Materials file.

## Abbreviations

The following abbreviations are used in this manuscript:

AIS	Adolescent Idiopathic Scoliosis
BMI	Body Mass Index
CI	Confidence Interval
I^2^	Inconsistency Index
MBrace	Mindfulness-Based Brace Intervention
NOS	Newcastle–Ottawa Scale
OR	Odds Ratio
PRISMA	Preferred Reporting Items for Systematic Reviews and Meta-Analyses
SD	Standard Deviation
SRS	Scoliosis Research Society
SOSORT	Society on Scoliosis Orthopaedic and Rehabilitation Treatment
